# Editorial: *South African Journal of Physiotherapy* 2018

**DOI:** 10.4102/sajp.v74i1.1320

**Published:** 2018-12-11

**Authors:** Aimee V. Stewart

**Affiliations:** 1 Department of Physiotherapy, School of Therapeutic Sciences, Faculty of Health Sciences, University of the Witwatersrand, South Africa

At the end of the year, we all tend to reflect on what the year has been like for us and we also consider what we may be planning for the year ahead. Part of my reflection this year has been to think about why the *South African Journal of Physiotherapy (SAJP)* is important and why it is worth putting in so much effort to continuously improve the standing of the journal. Many people are involved in producing our journal, and before going further, thanks need to be given to the Editorial Board, the reviewers, those who review with such good grace, the hard-working and supportive publishers and an increasingly broad readership. Do you read scientific journals all of you professional physiotherapists out there? Hope so!

We now are in the fortunate position of having many international physiotherapy journals, many of which are very good and publish cutting-edge and interesting findings that continue to propel our profession to constantly improve. Think about where we are now in our international professional standing and where we were not that long ago! So if there are so many of these journals, why is our journal important?

Our journal is important because we showcase our own research; we give a voice to our own researchers and have as our audience our own physiotherapy profession. We offer unique studies that are not considered by the large international journals that are mostly published in the developed world. We have unique and challenging health and rehabilitation issues that need not only to be aired but also to be considered by our profession. We need to constantly think about these unique challenges and how they can be managed by such an important rehabilitation profession as ours. We straddle the developed and developing world and because we have such a strong profession in South Africa and an increasingly strong group of researchers, we are in a position to start finding answers to the rehabilitation challenges that we and the rest of Africa experience. We have the means and the ability to consider and provide appropriate solutions to many of these challenges. We all need to encourage our researchers to do this; more and more researchers from other African countries, other developing countries and the developed world should join us in finding answers to the rehabilitation challenges faced by all of us.

As we move towards more and more of our physiotherapists in South Africa having PhDs, we must start considering large multi-centre trials that can start providing answers to some of our rehabilitation issues. We have worked hard to get to the point where we have this increasingly large cohort of established researchers and I call on them to do big studies and strengthen the *SAJP* by publishing their strong evidence to advance our profession here in South Africa. Increasingly, the *SAJP* must be one of the international journals of choice for our researchers. A process of inviting our top researchers and clinicians and/or researchers to contribute to our journal has begun with a favourable response from them.

How can we attract these researchers to publish their good studies in our journal? The only way to do so is by publishing good studies so that all our researchers can be encouraged to send their findings to our journal. This is a ‘chicken and egg’ scenario but, in reality, the only way that a journal improves. Getting onto international scientific lists of journals is a very important step and one that will encourage established researchers to publish in our journal; however, we must have good-quality articles to get there. The fact that we are now on PubMed is a huge step in the right direction. All who were involved in the arduous process to achieve this milestone must be congratulated!

We must now move towards the next steps in international recognition for the *SAJP* and I am sure with all who support and care for this journal, we can get to the point where our journal will be recognised as one of the many top international physiotherapy journals.

